# Efficacy of Alirocumab, Evolocumab, and Inclisiran in Patients with Hypercholesterolemia at Increased Cardiovascular Risk

**DOI:** 10.3390/medicina60071124

**Published:** 2024-07-12

**Authors:** Renata Rajtar-Salwa, Beata Bobrowska, Sylwia Socha, Artur Dziewierz, Zbigniew Siudak, Jakub Batko, Stanisław Bartuś, Agata Krawczyk-Ożóg

**Affiliations:** 1 Clinical Department of Cardiology and Cardiovascular Interventions, University Hospital, Macieja Jakubowskiego 2 Street, 30-688 Krakow, Polandartur.dziewierz@uj.edu.pl (A.D.);; 2 2nd Department of Cardiology, Jagiellonian University Medical College, Macieja Jakubowskiego 2 Street, 30-688 Krakow, Poland; 3 Collegium Medicum, Jan Kochanowski University in Kielce, 25-369 Kielce, Poland; 4 Department of Anatomy, Jagiellonian University Medical College, 12 Kopernika Street, 31-034 Krakow, Poland

**Keywords:** alirocumab, evolocumab, inclisiran, hypercholesterolemia, proprotein convertase subtilisin/kexin type 9 inhibitors

## Abstract

*Background and Objectives:* Lowering low-density lipoprotein (LDL-C) levels is critical for preventing atherosclerotic cardiovascular disease, yet some patients fail to reach the LDL-C targets despite available intensive lipid-lowering therapies. This study assessed the effectiveness and safety profile of alirocumab, evolocumab, and inclisiran in lipid reduction. *Materials and Methods:* A cohort of 51 patients (median (Q1–Q3) age: 49.0 (39.5–57.5) years) was analyzed. Eligibility included an LDL-C level > 2.5 mmol/L while on the maximum tolerated dose of statin and ezetimibe, a diagnosis of familial hypercholesterolemia, or a very high risk of cardiovascular diseases following myocardial infarction within 12 months prior to the study. Follow-ups and lab assessments were conducted at baseline (51 patients), 3 months (51 patients), and 15 months (26 patients) after the treatment initiation. *Results:* Median initial LDL-C levels 4.1 (2.9–5.0) mmol/L, decreasing significantly to 1.1 (0.9–1.6) mmol/L at 3 months and 1.0 (0.7–1.8) mmol/L at 15 months (*p* < 0.001). Total cholesterol also reduced significantly compared to baseline at both intervals (*p* < 0.001). No substantial differences in LDL-C or total cholesterol levels were observed between 3- and 15-month observations (*p* > 0.05). No statistically significant differences were noted in cholesterol reduction among the alirocumab, evolocumab, and inclisiran groups at 3 months. The safety profile was favorable, with no reported adverse cardiovascular events or significant changes in alanine transaminase, creatinine, or creatine kinase levels. *Conclusions:* Alirocumab, evolocumab, and inclisiran notably decreased LDL-C and total cholesterol levels without significant adverse effects, underscoring their potential as effective treatments in patients who do not achieve lipid targets with conventional therapies.

## 1. Introduction

The link between lipids, cholesterol, and atherosclerosis was already well established in the 1960s, catalyzing the development of lipid-lowering drugs [1]. Managing lipid disorders poses challenges, and achieving optimal low-density lipoprotein cholesterol (LDL-C) levels is crucial for both secondary and primary prevention of cardiovascular diseases. While statins are a cornerstone of lipid-lowering treatment, they often fall short of achieving target LDL-C levels or cause adverse effects in some patients [2]. A breakthrough in lipid-lowering therapy could be represented by proprotein convertase subtilisin/kexin type 9 (PCSK9) inhibitors (alirocumab and evolocumab) and inclisiran. Alirocumab and evolocumab are human monoclonal antibodies whose mechanism of action relates to the reduction in the plasma level of PCSK9 [3]. Inclisiran is a small interfering ribonucleic acid (siRNA) that inhibits the expression of PCSK9 by binding specifically to the mRNA precursor of PCSK9 protein and causing its degradation [2]. PCSK9 regulates the level of circulating LDL-C by enhancing the intracellular degradation of hepatic low-density lipoprotein receptors [2,3]. Its inhibition results in an increase in LDL receptors and a corresponding decrease in plasma LDL-C levels [4].

Familial hypercholesterolemia is an autosomal hereditary disorder characterized by hyper-LDL-C. A key gene associated with this condition is PCSK9 [5]. The prevalence of familial hypercholesterolemia is estimated to be between 1 in 200 and 1 in 250 individuals [6]. However, only a small fraction of these cases are identified and properly treated [7]. The most significant complication for individuals with familial hypercholesterolemia is coronary artery disease, due to systemic atherosclerosis. Additionally, patients with this condition also exhibit a higher prevalence of peripheral arterial disease and carotid atherosclerosis compared to those without. Therefore, early diagnosis, comprehensive treatment, and family screening are essential for effective management of familial hypercholesterolemia [8].

Current guidelines from the European Society of Cardiology (ESC) advocate lipid-lowering interventions in secondary prevention and very high-risk cardiovascular patients in primary prevention, aiming for an LDL-C level below 1.4 mmol/L (55 mg/dL) and a reduction of more than 50% from baseline [7,9]. However, particularly in patients with familial hypercholesterolemia, these therapeutic targets are frequently unmet [10]. Mutations in PCSK9-related genes are a key pathological mechanism in familial hypercholesterolemia, underscoring the potential benefit of PCSK9-targeted therapies [5]. These insights advocate a combined lipid-lowering strategy, integrating PCSK9 inhibitors or inclisiran, especially for patients inadequately managed with statin therapy alone. However, data on the application and effectiveness of this approach in real-life clinical scenarios are still limited.

### Aim

This retrospective study aims to evaluate the effectiveness and safety profile of alirocumab, evolocumab, and inclisiran in managing patients with familial hypercholesterolemia and/or patients at a very high risk of cardiovascular events, who have persistently high LDL-C levels despite the maximal tolerated dosages of statin and ezetimibe.

## 2. Material and Methods

### 2.1. Study Population

This study consisted of 51 consecutive patients treated with alirocumab, evolocumab, or inclisiran from October 2019 to November 2023 at the University Hospital in Krakow, Poland. The patients were diagnosed with familial hypercholesterolemia and/or classified as being at very high risk of cardiovascular events (according to point 3b in inclusion criteria). The inclusion criteria were as follows:

Age 18 years or older.
An LDL-C level greater than 100 mg/dL (2.5 mmol/L) despite adherence to a low-fat diet and:
  - Intensive treatment with statin at maximum doses for at least three months, which includes combination therapy with ezetimibe for at least one month. Here, ‘maximum doses’ refers to either atorvastatin 80 mg or rosuvastatin 40 mg when used in monotherapy. ‘Combination therapy’ refers to atorvastatin 40–80 mg or rosuvastatin 20–40 mg with ezetimibe 10 mg;
  - Or intensive statin therapy with the maximum tolerated doses for at least three months, including at least one month of combination therapy with ezetimibe 10 mg.
(a) A definitive diagnosis of heterozygous familial hypercholesterolemia, indicated by a score greater than 8 on the Dutch Lipid Clinic Network (DLCN) scale; (b) or, classification as a very high risk of cardiovascular events patient, defined as a patient with a documented myocardial infarction diagnosed invasively within the 12 months prior to enrollment, with either:
  - A history of additional myocardial infarction and multivessel coronary artery disease, defined as at least 50% stenosis in two or more vessels;
  - Or the presence of atherosclerotic disease in non-coronary arteries, such as peripheral artery disease or cerebrovascular disease (ischemic stroke or transient ischemic attack).

Patients who met the above criteria were enrolled in the study and received 150 mg alirocumab or 140 mg evolocumab subcutaneously every two weeks, or 284 mg of inclisiran via a single subcutaneous injection, administered initially, then after three months, and subsequently every six months. This regimen was in combination with a low-fat diet and the previously tolerated lipid-lowering treatment.

Exclusion criteria included secondary causes of hypercholesterolemia, homozygous familial hypercholesterolemia, a glomerular filtration rate below 30 mL/min/1.73 m^2^, advanced chronic liver disease classified as Child–Pugh class C, pregnancy, lactation, and hypersensitivity to the medications or their components. Moreover, severe allergic reactions following medication administration and ineffectiveness of treatment, assessed after three months as less than a 30% reduction in LDL-C levels relative to baseline, were grounds for patient exclusion from further treatment.

### 2.2. Data Collection

Baseline clinical data were collected retrospectively from patients’ medical records and through interviews conducted during their initial visit. Blood samples were taken at the time of enrollment and a follow-up of 3 months (51 patients) and 15 months (26 patients) post-initial medication dose. The parameters measured included LDL-C, total cholesterol, high-density lipoprotein cholesterol (HDL-C), triglycerides, alanine transaminase, creatine kinase, and creatinine levels.

The total cholesterol, triglycerides, and HDL-C levels were measured using the spectrophotometric method. Plasma LDL-C was calculated using the Friedewald formula: LDL-C = total cholesterol − HDL-C − (triglycerides/2.2) in mmol/L [7]. If the triglyceride level was >4.5 mmol/L, LDL-C was measured directly using enzymatic techniques. Alanine transaminase, creatine kinase, and creatinine levels were measured using enzymatic techniques. The measurements were conducted using a Roche Cobas Pro analyzer (Mannheim, Germany). During follow-up interviews, patients were assessed for any adverse effects linked to the medications and for the occurrence of cardiovascular events. A thorough medical interview was conducted, and all available medical documentation, including records of any outpatient visits or hospitalizations, was reviewed. There were no predefined criteria for the selection of medication among the three available options: alirocumab, evolocumab, and inclisiran. The choice of treatment depended on the doctor and its availability, and was not randomized.

### 2.3. Statistical Analysis

Statistical analysis was performed using SPSS 29.0 (Predictive Solutions, Pittsburgh, PA, USA). For continuous variables, data distribution was evaluated with the Shapiro–Wilk test and is reported as the median and lower and upper quartiles (Q1–Q3). Categorial variables are reported as numbers with percentages. The categorical variables were compared between groups using the chi square test. The continuous variables were compared between groups using the U-Mann Whitney test for sex comparison and using the Kruskal Wallis test for drugs comparison. Follow up comparisons were performed using the Wilcoxon signed-rank test. A *p*-value of less than 0.05 was considered statistically significant.

The study protocol adhered to the ethical guidelines of the 1975 Declaration of Helsinki. This study was approved by the Bioethics Committee of the Jagiellonian University, Cracow, Poland (No. 1072.6120.111.2021, date: 19 May 2021).

## 3. Results

The median (Q1–Q3) age of the patients included in the study was 49.0 (39.5–57.5) years, with men comprising 51.0% (26 patients) of the study population. Of all participants, 21 (41.2%) had a history of at least one myocardial infarction, 25 (49.0%) underwent percutaneous cardiac intervention, and 7 (13.7%) had coronary artery bypass grafting. Additionally, 8 (15.7%) patients had undergone peripheral angioplasty, 9 (17.6%) had diabetes mellitus, 23 (45.1%) suffered from arterial hypertension, 1 (2.0%) had atrial fibrillation, 5 (9.8%) had experienced a previous stroke, 8 (15.7%) were diagnosed with thyroid disorders, 2 (3.9%) had chronic kidney disease with a glomerular filtration rate > 30 mL/min/1.73 m^2^, and 20 (39.2%) were current or former smokers. Two patients (3.9%) had at least mild aortic stenosis on echocardiography. All patients had normal thyroid-stimulating hormone (TSH) levels, with a median value of 1.4 (1.1–2.0) µIU/mL. Clinical and demographic characteristics of each patient subgroup are detailed in Table 1. No significant differences were found between groups (*p* > 0.05).

The median baseline LDL-C level was 4.1 (2.9–5.0) mmol/L in the entire patient group, decreasing to 1.1 (0.9–1.6) mmol/L (*p* < 0.001) and 1.0 (0.7–1.8) mmol/L (*p* < 0.001) after 3 and 15 months following the initial dose of medication, respectively. The percentage reduction in LDL-C level was 71.8 (63.9–80.7) % after 3 months and 76.3 (52.0–84.7) % after 15 months. Median total cholesterol level at baseline was 6.2 (5.4–7.1) mmol/L, with significant reductions observed at both follow-up points: 2.9 (2.6–3.2) mmol/L (*p* < 0.001) after 3 months and 3.3 (2.5–4.1) mmol/L (*p* < 0.001) after 15 months. The percentage reduction in total cholesterol level compared to baseline was 49.7 (40.8–56.2) % after 3 months and 50.4 (34.2–59.7) % after 15 months. No statistically significant differences were observed between total cholesterol and LDL-C levels at these two time points (Table 2).

**Figure 1:**
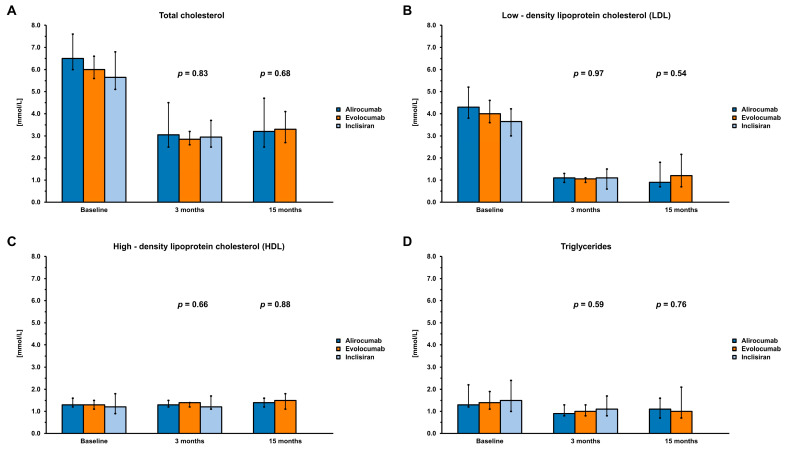
Blood laboratory test results at baseline and after 3 and 15 months of therapy. Panel (**A**)—Total cholesterol, Panel (**B**)—Low-density lipoprotein cholesterol, Panel (**C**)—High-density lipoprotein cholesterol, and Panel (**D**)—Triglycerides. Results presented as medians with 95% confidence intervals.

Comparisons between subgroups, distinguished by the medication taken (alirocumab, evolocumab, and inclisiran), showed no statistically significant differences in total cholesterol and LDL-C levels before and after 3 months of therapy (Table 3, Figure 1). A comparison after 15 months of therapy was conducted for alirocumab and evolocumab-treated groups due to the lack of patients in the inclisiran-treated group at that time (Table 4, Figure 1).

The therapeutic goal (LDL-C < 1.4 or <1.8 mmol/L) was achieved by 38 out of 51 patients (74.5%) after 3 months of therapy and 15 out of 26 patients (57.7%) after 15 months. In the alirocumab-treated group, the therapeutic goal was achieved by 76.0% of patients after 3 months and 60.0% of patients after 15 months. In the evolocumab-treated group, it was achieved by 72.2% and 54.5%, respectively. In the inclisiran-treated group, 75.0% of patients achieved the therapeutic goal after 3 months of therapy. No statistically significant difference was found in achieving therapeutic goals between the three drugs after 3 months (*p* = 0.96), or between evolocumab and alirocumab after 15 months (*p* = 0.77). No significant increases in the levels of alanine transaminase, creatinine, or creatine kinase were observed at either follow-up point. Moreover, no cardiovascular events were reported during the follow-up. Detailed data for all blood test results and comparisons are listed in Table 2, Table 3 and Table 4.

In the comparison between the two groups—patients with familial hypercholesterolemia (40 patients) and patients at a very high risk of cardiovascular events without familial hypercholesterolemia (11 patients)—we found that patients with familial hypercholesterolemia were younger. No differences were observed in total cholesterol, LDL-C, HDL-C, and triglyceride levels at any time points (Table S1), nor in achieving the therapeutic goal after 3 months (*p* = 0.25) and 15 months (*p* = 0.62).

Within the study cohort, four patients discontinued the therapy prematurely. Specifically, one patient in the alirocumab-treated group stopped therapy due to muscle and joint pain, despite no observed changes in creatinine or creatine kinase levels. Additionally, three patients (two from the alirocumab group and one from the evolocumab group) were excluded from ongoing therapy after three months due to insufficient LDL-C reduction (less than 30%).

## 4. Discussion

This analysis affirms the applicability and effectiveness of PCSK-9 targeted therapies in patients who do not achieve an LDL-C level reduction below 100 mg/dL (2.5 mmol/L) despite adhering to a low-fat diet and receiving intensive statin and ezetimibe therapy. The addition of alirocumab, evolocumab, or inclisiran to standard lipid-lowering regimens significantly reduces LDL-C and total cholesterol levels from baseline, as demonstrated by the follow-up data.

LDL-C is a widely recognized and modifiable risk factor for cardiovascular disease. Numerous studies have demonstrated its role in the development of atherosclerosis, emphasizing the importance of reducing LDL-C using any means available [11]. Statins and ezetimibe are often sufficient for achieving target LDL-C levels in cardiovascular patients when prescribed in high doses, such as 40 mg rosuvastatin, 80 mg of atorvastatin, and 10 mg of ezetimibe. In instances where this therapy does not adequately reduce LDL-C, an available option is the use of monoclonal antibodies that inhibit PCSK9, which are a new class of drugs that effectively lower LDL-C levels [12,13]. Additionally, inclisiran, a pioneering cholesterol-lowering siRNA, has been approved for use in adults with primary hypercholesterolemia (both heterozygous familial and non-familial) or mixed dyslipidemia, as an adjunct to dietary measures. It is intended for use in combination with statins and other lipid-lowering therapies in patients who cannot achieve their LDL-C targets with the maximum tolerated dose of statins [14].

In the FOURIER trial, a double-blind, placebo-controlled study, 27,564 patients with atherosclerotic cardiovascular disease and LDL-C levels of 70 mg/dL (1.8 mmol/L) or higher, who were already on statin therapy, were enrolled [15]. These patients were randomly assigned to receive evolocumab (either 140 mg every 2 weeks or 420 mg monthly) or a matching placebo through subcutaneous injections. Of these, 13,784 patients received evolocumab. In the evolocumab group, 69.5% of these patients were on high-intensity statin therapy. At 48 weeks, the evolocumab group showed a least-squares mean percentage reduction in LDL-C of 59% compared to the placebo group (*p* < 0.001), which translates to a mean absolute reduction of 56 mg/dL (1.45 mmol/L), reaching a median of 30 mg/dL (0.78 mmol/L). The median follow-up duration was 2.2 years.

In our study, 18 patients (35.3%) were treated with evolocumab in combination with other lipid-lowering agents, such as atorvastatin in 4 (22.2%) patients and rosuvastatin in 11 (61.1%) patients, with all patients also receiving ezetimibe. Among this group, we observed statin intolerance in 3 (16.7%) patients.

After 3 months of treatment with evolocumab, a median 69.8 (62.1–78.0) % reduction in LDL-C was noted. While previous studies have shown that evolocumab can reduce LDL-C levels by approximately 60% [16,17], our clinical practice observations have identified a similar reduction in LDL-C levels.

In addition, in the GLAGOV study, a randomized, double-blind, placebo-controlled trial, 968 patients with symptomatic coronary artery disease were treated with either statins alone or in combination with evolocumab [18]. The study evaluated changes in percent atheroma volume and the regression of coronary atheroma. The addition of evolocumab to statin therapy resulted in achieving mean LDL-C levels of 36.6 mg/dL and led to atheroma regression with a mean change in percent atheroma volume of approximately 1% (*p* < 0.001), with a greater percentage of patients experiencing regression.

Huang and colleagues conducted a meta-analysis of 22 studies involving 42,786 patients [19]. They found that the addition of PCSK9 inhibitors to statin therapy resulted in LDL-C redactions of 50–63% across all studies. Specifically, evolocumab 140 mg was identified as one of the most effective strategies for lowering LDL-C, with a treatment difference in LDL-C reduction of 68.05% (95% confidence interval (CI), 62.43% to 73.67) compared to a placebo. The study also found no significant difference in the risk of adverse events between PCSK9 inhibitors.

In a meta-analysis by Imran and colleagues, 54 studies involving 87,669 participants were analyzed [20]. LDL-C percent change was reported in 47 studies (n = 62,634) evaluating two PCSK9 inhibitors and siRNA therapy. Of those, 21 studies (n = 41,361) involved treatment with evolocumab (140 mg), 22 (n = 11,751) involved alirocumab (75 mg), and 4 studies (n = 9522) involved inclisiran (284 mg and 300 mg). Compared to a placebo, after a median of 24 weeks (IQR 12–52), evolocumab reduced LDL-C by −61.09% (95% CI: −64.81 to −57.38, *p* < 0.001), highlighting its efficacy in significantly lowering LDL-C levels by more than 40% in high-risk individuals. Furthermore, both alirocumab and evolocumab were associated with a reduced risk of major adverse cardiovascular events, with alirocumab also showing a reduction in cardiovascular and all-cause mortality.

One pivotal study demonstrating the effectiveness of alirocumab treatment is the ODYSSEY OUTCOMES study. This multicenter, randomized, double-blind, placebo-controlled trial involved 18,924 patients post-acute coronary syndrome, all intensively treated with statins. It assessed the additional impact of alirocumab compared to a placebo over a median follow-up of 2.8 years on cardiovascular event risk. Alirocumab led to a 15% reduced risk of the primary composite endpoint, and except for a higher incidence of local post-injection reactions (3.8% vs. 2.1%), adverse events were similar across both groups. In the alirocumab cohort, the mean LDL-C level decreased to 40 mg/dL (1.0 mmol/L) and 48 mg/dL (1.2 mmol/L) after 4 and 12 months, respectively—reflecting a reduction of approximately 57% and 48% from baseline values [21]. In comparison, our study showed a more substantial reduction in LDL-C: the median reduction in LDL-C level in the alirocumab-treated group was about 74% and 85% after 3 and 15 months, respectively. Notably, the initial LDL-C levels of our patients were higher (alirocumab-treated group: mean 4.4 ± 1.2, median 4.3 (3.4–5.2) mmol/L) than those in the ODYSSEY OUTCOMES study (2.38 ± 0.80 mmol/L). Interestingly, researchers in Israel reported a lower reduction, observing a 31.7% decrease in LDL-C among 623 patients treated with alirocumab through a national health provider [22]. The reduced efficacy may be attributed to nearly half of the patients (47%) discontinuing their background statin and/or ezetimibe therapy, resulting in 65% (n = 407) being treated solely with alirocumab during the follow-up of 120 ± 60 days. Conversely, in our study and the ODYSSEY OUTCOMES study, patients maintained intensive statin therapy, often combined with ezetimibe, which yielded LDL-C reductions. In addition, a Japanese study of nearly 1200 patients on optimal statin treatment with or without ezetimibe documented a LDL-C reduction of around 50% [23]. Here, the mean LDL-C levels decreased from 119.9 ± 49.3 mg/dL to 59.7 ± 40.2 mg/dL by week 4 and remained consistent through weeks 52 and 104. The respective mean and percentage change in LDL-C levels from baseline to the last observation carried forward was −59.8 ± 43.8 mg/dL and −46.9 ± 2.1% in the non-familial hypercholesterolemia group, and −59.1 ± 51.5 mg/dL and −42.7 ± 2.0% in the familial hypercholesterolemia group, confirming varied individual responses to alirocumab. Our study did not report any significant cardiovascular events or alirocumab-related events leading to therapy discontinuation. Moreover, the proportion of patients achieving their target LDL-C level was satisfactory, with 76.0% after 3 months and 60.0% after 15 months in alirocumab-treated group. These findings align with Banach’s post hoc analysis of the ODYSSEY APPRISE study, comprising 994 patients, where a higher percentage of patients achieved LDL-C levels < 1.8 mmol/L and/or ≥50% reduction from baseline when adhering strictly to alirocumab therapy, underlining the treatment’s safety and efficacy [24].

Inclisiran and PCSK9 inhibitors both target PCSK9, but their mechanisms differ significantly. Inclisiran operates through RNA interference, utilizing siRNAs to inhibit gene transcription [25]. This therapy specifically inhibits the production of the PCSK9 protein in the liver, which results in the lowering of LDL-C levels. Clinical trials have consistently confirmed its efficacy. For instance, in the ORION-1 study, a single 300 mg dose of inclisiran was shown to decrease LDL-C by 38.4% at 180 days post-administration, and a two-dose regimen resulted in a 52.6% reduction [26], with these decreases being maintained over time [27]. The Phase III ORION-9, 10, and 11 trials further demonstrated that inclisiran consistently reduced LDL-C by 39.7% to 51.3% on the 510th day compared to a placebo, in addition to lowering non-HDL-C, apolipoprotein B, triglyceride, and lipoprotein (a) levels [28]. While direct comparisons with PCSK9 inhibitors are not yet available, network meta-analyses offer some insight. One analysis of 22 randomized controlled trials in patients with hypercholesterolemia on statin treatment found that inclisiran reduced LDL-C by an average of 50.08% compared to a placebo, a result comparable to the 54.64% reduction with alirocumab and slightly less than the 63.41% reduction with evolocumab [19]. Another meta-analysis of 48 studies corroborated these findings, indicating that inclisiran can halve LDL-C levels irrespective of whether patients are on statin therapy, have atherosclerotic cardiovascular disease, or have familial hypercholesterolemia [29]. These results are in line with our findings. In terms of safety, inclisiran has been generally well tolerated, with most adverse effects being mild to moderate reactions at the injection site [19,28,29]. A potential benefit of inclisiran over PCSK9 inhibitors is its dosing frequency: it requires injections every three to six months, thereby providing a more extended duration of action. In contrast, PCSK9 inhibitors typically require more frequent administration, with injections needed once or twice a month [25].

PCSK9 monoclonal antibodies modestly reduce plasma triglyceride levels, primarily by enhancing low-density lipoprotein receptor-mediated catabolism of intermediate-density lipoproteins [30]. In our study, triglyceride levels were significantly reduced after 3 months of therapy (*p* < 0.001). However, the reductions observed after 15 months were not significant (*p* = 0.08). These reductions are attributed to both the new medication and patient education on controlled diets during medical visits. The difference in significance of triglyceride level reduction after 15 months may be due to patients becoming less adherent to their diet or could be coincidental.

The study faced several limitations. It was a retrospective and observational study. The sample size was small, with a particularly limited number of patients meeting the criteria for treatment reimbursement. Furthermore, the intended 15-month follow-up data were not available for all patients, as some underwent shorter durations of treatment. Additionally, lipoprotein (a) levels were not controlled during the follow-up period. However, despite these constraints, the study provides preliminary results that may support the increasing recognition of PCSK9 inhibitors and inclisiran as a novel and efficacious treatment option for patients with familial hypercholesterolemia or those at very high risk of cardiovascular diseases.

## 5. Conclusions

In patients with familial hypercholesterolemia or those at very high cardiovascular risk, the adjunctive use of alirocumab, evolocumab, or inclisiran to maximum tolerated doses of statin and ezetimibe resulted in a substantial LDL-C reduction during the follow-up period. Comparative analysis of the drugs revealed no statistically significant differences in total cholesterol and LDL-C levels before and after 3 months of treatment. Moreover, no adverse cardiovascular events or significant changes in creatine kinase, alanine transaminase, or creatinine levels were observed throughout the study period.

## Figures and Tables

**Table 1:** Baseline characteristics.

		Alirocumab Users (*n* = 25)	Evolocumab Users (*n* = 18)	Inclisiran Users (*n* = 8)	*p*-Value
Age, year, median (Q1–Q3)		54.0 (44.0–59.0)	48.0 (38.0–57.0)	41.0 (33.0–51.0)	0.12
Male, N (%)		11 (44.0%)	10 (55.6%)	5 (62.5%)	0.59
BMI, kg/m^2^, median (Q1–Q3)		25.8 (23.4–27.7)	25.5 (22.7–29.7)	26.8 (24.7–29.6)	0.70
Previous myocardial infarction, N (%)		13 (52.0%)	5 (27.8%)	3 (37.5%)	0.27
Previous percutaneous coronary intervention, N (%)		14 (56.0%)	8 (44.4%)	3 (37.5%)	0.59
Previous coronary artery bypass grafting, N (%)		6 (24.0%)	0 (0.0%)	1 (12.5%)	-
Arterial hypertension, N (%)		12 (48.0%)	9 (50.0%)	2 (25.0%)	0.46
Diabetes mellitus type II, N (%)		4 (16.0%)	4 (22.2%)	1 (12.5%)	0.80
Atrial fibrillation, N (%)		1 (4.0%)	0 (0.0%)	0 (0.0%)	-
Thyroid disorders, N (%)		4 (16.0%)	3 (16.7%)	1 (12.5%)	0.96
TSH, µIU/mL, median (Q1–Q3)		1.5 (1.1–2.0)	1.5 (1.1–2.5)	1.2 (1.1–1.4)	0.56
Chronic kidney disease, N (%)		2 (8.0%)	0 (0.0%)	0 (0.0%)	-
Pulmonic disease, N (%)		1 (4.0%)	0 (0.0%)	0 (0.0%)	-
Previous transient ischemic attack and/or stroke, N (%)		2 (8.0%)	3 (16.7%)	0 (0.0%)	-
Ever smoker, N (%)		10 (40.0%)	5 (27.8%)	5 (62.5%)	0.25
At least mild aortic stenosis in echocardiography N (%)		1 (4.0%)	1 (5.6%)	0 (0.0%)	-
	Atorvastatin	3 (12.0%)	4 (22.2%)	0 (0.0%)	0.27
Statins, N (%)	Rosuvastatin	14 (56.0%)	11 (61.1%)	8 (100.0%)	
	Pitavastatin	1 (4.0%)	0 (0.0%)	0 (0.0%)	
	Intolerance	7 (28.0%)	3 (16.7%)	0 (0.0%)	
Lipoprotein (a), g/L, median (Q1–Q3)		0.5 (0.2–1.4)	0.8 (0.3–1.0)	0.2 (0.0–0.4)	0.09

BMI—Body Mass Index, N—number of patients, TSH—thyroid-stimulating hormone, Q1–Q3, lower and upper quartiles.

**Table 2:** Blood laboratory test results at baseline and during follow-up.

	Baseline (N = 51)	3 Months (N = 51)	*p*-Value (Baseline vs. 3 Months)	15 Months (N = 26)	*p*-Value (Baseline vs. 15 Months)	*p*-Value (3 Months vs. 15 Months)
Total cholesterol, mmol/L	6.2 (5.4–7.1)	2.9 (2.6–3.2)	<0.001	3.3 (2.5–4.1)	<0.001	0.07
LDL-C, mmol/L	4.1 (2.9–5.0)	1.1 (0.9–1.6)	<0.001	1.0 (0.7–1.8)	<0.001	0.31
HDL-C, mmol/L	1.3 (1.1–1.5)	1.3 (1.2–1.5)	0.51	1.5 (1.1–1.7)	0.59	0.90
Triglyceride, mmol/L	1.4 (1.1–2.0)	1.0 (0.8–1.4)	<0.001	1.0 (0.7–1.6)	0.08	0.18
Alanine transaminase, U/L	37.0 (22.0–52.0)	33.0 (22.0–52.0)	0.11	34.0 (22.0–45.0)	0.34	0.77
Creatine kinase, U/L	97.0 (82.0–197.0)	104.0 (81.0–152.0)	0.42	141.0 (105.0–195.0)	0.14	0.10
Creatinine, µmol/L	77.2 (63.6–82.3)	77.1 (65.6–84.5)	0.95	80.9 (67.7–89.7)	0.63	0.68

N—number of patients; LDL-C—low-density lipoprotein cholesterol; HDL-C—high-density lipoprotein cholesterol. Results presented as median and lower and upper quartiles (Q1–Q3).

**Table 3:** Blood laboratory test results at baseline and after 3 months of therapy.

		Baseline	3 Months	*p*-Value (Baseline vs. 3 Months)	*p*-Value (Alirocumab vs. Evolocumab vs. Inclisiran) after 3 Months of Therapy
Reduction in LDL-C level (%)	Alirocumab	-	73.5 (63.4–81.7)	-	0.80
	Evolocumab	-	69.8 (62.1–78.0)	-	
	Inclisiran	-	71.5 (67.4–76.7)	-	
Reduction in total cholesterol level (%)	Alirocumab	-	50.0 (38.8–61.3)	-	0.84
	Evolocumab	-	48.9 (40.9–56.1)	-	
	Inclisiran	-	48 (44.3–51.5)	-	
Total cholesterol, mmol/L	Alirocumab	6.5 (5.9–7.7)	3.1 (2.5–5.0)	<0.001	0.83
	Evolocumab	6.0 (5.4–7.1)	2.9 (2.6–3.2)	<0.001	
	Inclisiran	5.7 (5.1–6.7)	3 (2.6–3.7)	<0.001	
LDL-C, mmol/L	Alirocumab	4.3 (3.4–5.2)	1.1 (0.8–1.3)	<0.001	0.97
	Evolocumab	4.0 (2.9–5.0)	1.1 (0.9–1.6)	<0.001	
	Inclisiran	3.7 (3.2–4.2)	1.1 (0.7–1.5)	<0.001	
HDL-C, mmol/L	Alirocumab	1.3 (1.1–1.5)	1.3 (1.2–1.6)	0.65	0.66
	Evolocumab	1.3 (1.1–1.5)	1.4 (1.2–1.5)	0.32	
	Inclisiran	1.2 (1.1–1.7)	1.2 (1.1–1.5)	0.73	
Triglyceride, mmol/L	Alirocumab	1.3 (0.8–2.3)	0.9 (0.7–1.4)	0.003	0.59
	Evolocumab	1.4 (1.1–2.0)	1.0 (0.7–1.4)	<0.001	
	Inclisiran	1.5 (1.1–2.2)	1.1 (0.9–1.6)	0.012	
Alanine transaminase, U/L	Alirocumab	28.0 (21.0–43.0)	25.0 (21.0–35.0)	0.88	0.06
	Evolocumab	37.0 (22.0–52.0)	43.0 (30.0–57.0)	0.07	
	Inclisiran	30.0 (21.0–55.0)	33.0 (27.0–56.0)	0.11	
Creatine kinase, U/L	Alirocumab	108.0 (91.0–157.0)	107.0 (75.0–164.0)	0.82	0.80
	Evolocumab	97.0 (82.0–197.0)	103.0 (84.0–125.0)	0.42	
	Inclisiran	106.0 (83.0–190.0)	91.0 (67.0–152.0)	0.74	
Creatinine, µmol/L	Alirocumab	73.0 (65.0–87.4)	81.3 (65.6–90.7)	0.39	0.57
	Evolocumab	77.2 (63.6–82.3)	72.1 (64.7–82.6)	0.53	
	Inclisiran	77.7 (59.3–79.7)	78.5 (71.7–82.4)	0.89	

N—number of patients; LDL-C—low-density lipoprotein cholesterol; HDL-C—high-density lipoprotein cholesterol. Results presented as median and lower and upper quartiles (Q1–Q3).

**Table 4:** Blood laboratory test results at baseline and after 15 months of therapy.

		Baseline	15 Months	*p*-Value (Baseline vs. 15 Months)	*p*-Value (Alirocumab vs. Evolocumab) after 15 Months of Therapy
Reduction in LDL-C level (%)	Alirocumab	-	85.4 (55.6–86.0)	-	0.80
	Evolocumab	-	56.3 (48.2–71.6)	-	
Reduction in total cholesterol level (%)	Alirocumab	-	54.8 (44.5–64)	-	0.41
	Evolocumab	-	42.7 (23.4–62)	-	
Total cholesterol, mmol/L	Alirocumab	6.5 (5.9–7.7)	3.2 (2.5–4.7)	<0.001	0.68
	Evolocumab	6.0 (5.4–7.1)	3.3 (2.7–4.1)	<0.001	
LDL-C, mmol/L	Alirocumab	4.3 (3.4–5.2)	0.9 (0.6–1.8)	<0.001	0.54
	Evolocumab	4.0 (2.9–5.0)	1.2 (0.7–2.2)	<0.001	
HDL-C, mmol/L	Alirocumab	1.3 (1.1–1.5)	1.4 (1.2–1.6)	0.842	0.80
	Evolocumab	1.3 (1.1–1.5)	1.5 (1.1–1.8)	0.594	
Triglyceride, mmol/L	Alirocumab	1.3 (0.8–2.3)	1.1 (0.7–1.6)	0.140	0.76
	Evolocumab	1.4 (1.1–2.0)	1.0 (0.7–2.1)	0.248	

N—number of patients; LDL-C—low-density lipoprotein cholesterol; HDL-C—high-density lipoprotein cholesterol Results presented as median and lower and upper quartiles (Q1–Q3).

## Data Availability

The data from the study are available upon reasonable request from the corresponding author.

## Supplementary Materials

The following supporting information can be downloaded at: https://www.mdpi.com/article/10.3390/medicina60071124/s1, Table S1. Baseline characteristics and cholesterol blood test results in patients at very high risk of cardiovascular events patient and patients with familial hypercholesterolemia.
