# Supplementary material for: Efficacy of Alirocumab, Evolocumab, and Inclisiran in Patients with Hypercholesterolemia at Increased Cardiovascular Risk
Source: Medicina (Kaunas). 2024 Jul 12;60(7):1124. doi: 10.3390/medicina60071124 (PMC11278919; doi:10.3390/medicina60071124)
Supplement: Supplementary file 1 [file medicina-60-01124-s001.zip › medicina-3077655-supplementary.pdf]

Table S1. Baseline characteristics and cholesterol blood test results in patients at very high risk of cardiovascular events patient and patients with familial hypercholesterolemia

	Very high risk of cardiovascular events (N=11)	Familial hypercholesterolemia (N=40)	P-value
Age, year	56.0 (47.0-65.0)	47.5 (36.5-55.0)	**0.04**
BMI, kg/m^2	26.4 (24.6-27.7)	25.7 (23.4-29.7)	0.96
Previous myocardial infarction, N (%)	11 (100%)	10 (25.0%)	**<0.001**
Previous percutaneous coronary intervention, N (%)	10 (90.9%)	15 (37.5%)	**0.002**
Previous coronary artery bypass grafting, N (%)	1 (9.1%)	6 (15.0%)	1.00
Arterial hypertension, N (%)	8 (72.7%)	15 (37.5%)	0.05
Diabetes mellitus type II, N (%)	1 (9.1%)	8 (20%)	0.66
Atrial fibrillation, N (%)	0 (0%)	1 (2.5%)	-
Thyroid disorders, N (%)	1 (9.1%)	7 (17.5%)	0.83
TSH, μIU/mL	1.39 (1.1-1.8)	1.46 (1.1-2.1)	0.90
Chronic kidney disease, N (%)	1 (9.1%)	1 (2.5%)	0.36
Pulmonic disease, N (%)	1 (9.1%)	0 (0%)	-
Previous transient ischemic attack and/or stroke, N (%)	0 (0%)	5 (12.5%)	0.57
Ever smoker, N (%)	6 (54.5%)	14 (35%)	0.30
At least mild aortic stenosis in echocardiography N (%)	0 (0%)	2 (5.0%)	-
Lipoprotein (a), g/L	0.95 (0.48-1.42)	0.52 (0.18-1.25)	0.43
**Baseline**			
Total cholesterol, mmol/L	5.4 (4.8-6.7)	6.4 (5.7-7.45)	0.16
LDL-C, mmol/L	3.6 (2.8-4.3)	4.2 (3.4-5.05)	0.22
HDL-C, mmol/L	1.3 (1.1-1.4)	1.3 (1.1-1.6)	0.54
Triglyceride, mmol/L	1.4 (1.2-2.0)	1.4 (0.9-2.3)	0.83
**3 months**			

Total cholesterol, mmol/L	2.9 (2.5-3.3)	2.9 (2.5-4.0)	0.88
LDL-C, mmol/L	1.1 (0.8-1.3)	1.1 (0.8-1.7)	0.38
HDL-C, mmol/L	1.3 (1.2-1.4)	1.3 (1.2-1.6)	0.73
Triglyceride, mmol/L	1.3 (1.0-1.6)	0.9 (0.7-1.4)	0.13
**15 months**			
Total cholesterol, mmol/L	3.6 (3.5-4.9)	3.0 (2.5- 4.1)	0.28
LDL-C, mmol/L	1.5 (0.8- 1.7)	1.0 (0.7- 1.8)	0.67
HDL-C, mmol/L	1.3 (1.2- 1.4)	1.5 (1.1- 1.7)	0.54
Triglyceride, mmol/L	1.4 (1.3-2.0)	0.9 (0.7-1.5)	0.20

BMI - body mass index, N - number of patients, TSH - thyroid-stimulating hormone, LDL-C - low-density lipoprotein cholesterol, HDL-C - high-density lipoprotein cholesterol. Results presented as median and lower and upper quartiles (Q1-Q3).
